# Supporting Self-Regulated Learning in Distance Learning Contexts at Higher Education Level: Systematic Literature Review

**DOI:** 10.3389/fpsyg.2021.792422

**Published:** 2022-01-18

**Authors:** Natalia Edisherashvili, Katrin Saks, Margus Pedaste, Äli Leijen

**Affiliations:** Institute of Education, University of Tartu, Tartu, Estonia

**Keywords:** self-regulated learning, support interventions, distance learning, higher education, systematic review

## Abstract

Shifting learning to distant formats especially at the higher education level has been unprecedented during the past decade. Diverse digital learning media have been emerging which allow learner autonomy, and at the same time, require the ability of efficient regulation of various aspects of the learning process for sustainable academic progress. In this context, supporting students in self-regulated learning (SRL) in an optimal way becomes an important factor for their academic success. The present study attempts through a systematic review of 38 studies to provide an overview of the interventions identified as supporting all areas of SRL (metacognitive, cognitive, motivational and emotional), in its three phases (preparatory, performance, appraisal) in distance education environments at the higher education level. As the study results show, there are a number of SRL support interventions available with proven positive effect on SRL. However, their distribution has been found to be uneven. Whereas metacognition regulation and the performance phase of learning is vastly investigated, the emotion regulation, and the preparatory and appraisal phases of the SRL cycle are somewhat underexplored. As complex and multi-component as the process of SRL is, the combination of various interventions, and specific features, for more comprehensive support has also been found beneficial. Additionally, it has been revealed that the emotion regulation, in most cases, is closely related to motivation regulation, and similar interventions support these two. Future studies can further explore the efficiency and relevance of the identified interventions, taking closer look at the effects of various digital media, learner characteristics as well as different levels of education on learners’ self-regulation needs.

## Introduction

Digitalization and shifting to distant modes of operation have affected the 21st century living, studying and working dramatically. However, the start of the COVID-19 pandemic triggered even stronger, irreversible reliance on digital technologies, in general, and the largest “online movement” in the history of education, in particular. The current tendencies in the field of education indicate that operation in the distance learning environment is becoming more and more common and will eventually turn into a new normal beyond the era of the COVID-19 pandemic. Consequently, relevant adaptations and finding new ways to cope with the new reality in the field of education are emerging (Chick et al., 2020; Daniel, 2020). In the field of education, the shift to distance learning is especially visible and more widely adopted at the higher education level. At this stage, students are already expected to have some degree of competence to function independently, and thus, as long as the proper instructional design is put in place, and learners are further supported in their self-regulated learning needs, the chances of success become feasible (Broadbent and Poon, 2015).

In this era of unprecedented digitalization and distance learning, reconsideration of the ways that have been used to support learners in their study process has become crucial. Even though self-regulated learning (SRL) is relevant for face-to-face (F2F) learning formats as well, it is distance learning that makes the importance of SRL more pronounced (Breslow et al., 2013; Jordan, 2014). In the absence of the instructor’s direct supervision and guidance witnessed in many distance learning formats, the importance of SRL becomes even more crucial and a determining factor for the successful implementation of the learning process and learners’ improved academic outcomes (Veenman, 2011; Broadbent and Poon, 2015; Wong et al., 2019). Unlike some decades ago, when learning technologies were just environments where highly structured information was presented electronically, today, learners have to actively get involved in planning their own learning paths, setting their goals, using the best strategies to get to those goals, monitoring their progress, reflecting upon their learning and adapting accordingly (Carter et al., 2020). It has been demonstrated that the lack of ability to self-regulate and operate efficiently under new education norms and circumstances is causing learning difficulties for students (Baticulon et al., 2021). A huge discrepancy between the enrollment and completion rates in Massive Open Online Courses (MOOCs), for instance, is a further indication of the importance of the support learners need in the process of distance learning (Breslow et al., 2013).

Self-regulated learning becomes even more important at the university level, when studying becomes more intense and complex (Khiat, 2019). Higher education was heading toward the shift to distance learning even before the pandemic, and at present, does so in a more accelerated manner. Additionally, it is at the higher education level where the distance learning model is expected to hold most extensively beyond the COVID-19 pandemic (Gallagher and Palmer, 2020).

Further, engaging in self-regulated learning in an efficient manner, in a digital environment which is open-ended, non-linear and information-rich (Duffy and Azevedo, 2015), and especially in the ones characterized by “massiveness and heterogeneity of the participants” (Pérez-Álvarez et al., 2018, p. 17), does not happen automatically. It is claimed that learners’ SRL competence should be developed and supported in a targeted way (Panadero and Alonso Tapia, 2014). Thus, the selection of the SRL support mechanisms has to be conducted in a thorough manner, bearing in mind that the provided help should not only technically support the learner in the given learning context, making them reliant on the given support mechanisms, but rather be conducive to the development of learners’ transferable SRL skills.

## Background

### Self-Regulated Learning

**FIGURE 1:**
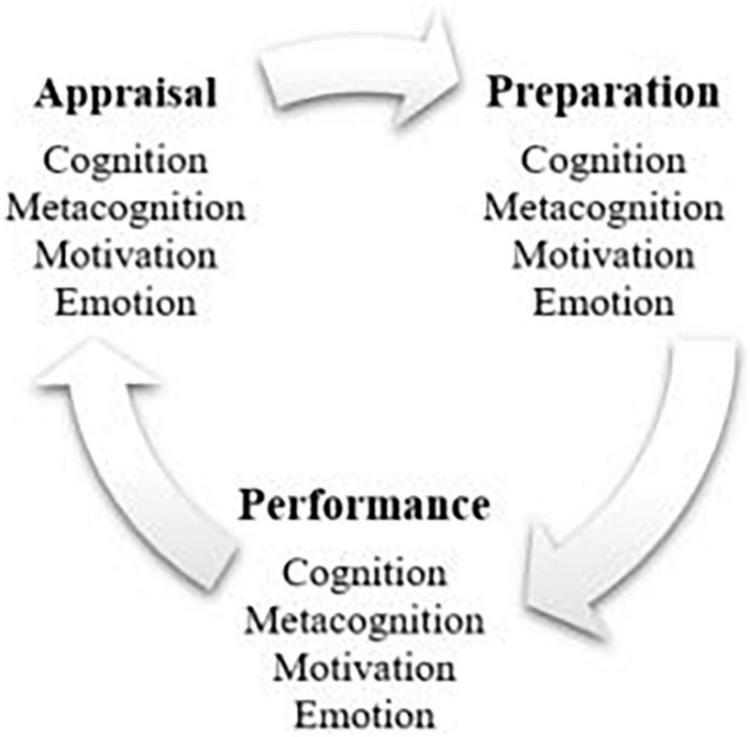
Cyclical model of Self-regulated learning (SRL) adapted from Panadero (2017).

Over the past two decades, SRL has become one of the major areas of research in educational psychology, and it is often referred to as the driving competence needed for transforming individuals into successful independent learners (Boekaerts, 1999). SRL has been widely explored by many authors, proposing various angles of seeing the process. What the existing different models of SRL have in common, though, is its cyclical, multi-phase and multi-component nature (Panadero and Alonso Tapia, 2014). The SRL model adopted in the current review (see Figure 1) is based on the version suggested by Panadero (2017), and is also applied in the study by Hooshyar et al. (2020).

The given model is detailed enough to cover all aspects of SRL: all three of its phases (preparation, performance and appraisal) as well as the areas (cognition, metacognition, motivation and emotion). Exploration of SRL along these lines will allow a comprehensive analysis of the whole process and identification of the targeted support interventions.

### Self-Regulation and Distance Learning

As repeatedly witnessed during COVID-19 pandemics, where the emergency shift toward online learning became a necessity at each level of education, succeeding academically in distant contexts requires a different set of skills on learners’ part from the ones they were used to in f2f learning formats (Shnaubert and Herold, 2020). In f2f context, the presence of the teacher helps learners throughout all phases of learning — planning and preparation; monitoring and supporting of the learning process through close observation and just-in-time feedback provision. Additionally, the physical presence of the instructor as well as peers has been found to be conducive to transforming learning into emotionally and motivationally more accommodating process. It is exactly emotions and motivation that have been found particularly challenging to support in the distance learning formats, requiring special adaptations and instructional design planning (Shnaubert and Herold, 2020). Thus, the SRL skills needed in f2f and distant environment cannot be equated, and should be investigated separately.

### Self-Regulated Learning at Higher Education Level

Another important factor to be considered while narrowing down the scope of SRL research is the education level. Learners of different ages differ considerably by the way they learn – the methods that help them prepare for their studies, the ways they process information, stay focused and motivated. The factors that contribute to the initiation and support of SRL learning also differ across different age groups. If in case of children and school contexts it is the teacher who plays the defining role for learners’ successful SRL, in case of adults, the instructional design is seen to be a driving factor (Oates, 2019). Also, whereas it is implicit approaches that have been found to be working for developing SRL skills in case of young learners (Wagener, 2013), more explicit interventions have been found helpful for adults (Raaijmakers et al., 2018). Academic demands and expectations also differ across the age groups (Taylor and Hamdy, 2013; Kellenberg et al., 2017). at the higher education level, learners are expected to be more autonomous, and in need of taking control of their own learning process (Zimmerman, 2000; Dillon and Greene, 2003), while also dealing with more “high stakes” tasks (e.g., tests, interviews, preparing for the job), and require specific SRL skills to be able to effectively manage one’s learning behavior as well as motivation and emotions (Shnaubert and Herold, 2020). Anxiety levels involved in the learning process have been found to be higher in case of adult learners, calling for more attention toward identifying adequate and sustainable supporting measures in this regard (Kellenberg et al., 2017).

### Previous Research and an Existing Gap

For the past decade, an increasing number of literature reviews has been conducted on the interventions supporting SRL in online learning. The focus of the research available has varied, however. Some studies have focused on identifying as many concrete tools/platforms supporting SRL as possible without particularly checking their actual impact (Pérez-Álvarez et al., 2018; Yen et al., 2018) on learners’ abilities or performance. Some have aimed at providing focus on one particular type of online learning environment (Pérez-Álvarez et al., 2018) or how to support a particular area (metacognition and its specific aspects) of SRL (Dabbagh and Kitsantas, 2004); others have centered around the efficiency of concrete interventions, such as prompts, feedback and/or their combination (Wong et al., 2019), around concrete technological features (Yen et al., 2018), specific study domains (Devolder et al., 2012) or concrete types of tasks (Azevedo et al., 2011). Other studies have reviewed SRL support interventions during a concrete period of time (Tsai, 2013) or focused on the design recommendations of SRL support interventions (Pérez-Álvarez et al., 2016). Yet more recent studies have looked at how SRL measurement tools can be employed for SRL support purposes, a new interesting trend in measuring and supporting SRL simultaneously (Panadero et al., 2015; Araka et al., 2020). The potential of the measurement and support of SRL, the Open Learner Model (Hooshyar et al., 2020) and Learning Analytics (Marzouk et al., 2016; Matcha et al., 2020) have also been explored. However, there are missing reviews focusing on the effects of interventions on different phases and areas of SRL comprehensively and systematically.

### The Aim of the Current Study

It was deemed important to keep the scope of the study focused, and explore SRL in the context of distance learning at the higher education level (see also Section “Further Research and Limitations” on the study limitations). Such approach facilitates capturing the specific nature of the given contexts comprehensively (see section “Self-Regulation and Distance Learning” and “Self-Regulation and Higher Education Level” above), and making the findings context-specific and readily applicable.

Thus, the current study investigates how SRL can be best supported in distance learning environments at higher education level. It aims to identify the interventions, or combination of interventions, that have been found to have a demonstrated effect on supporting each area of SRL at each of its phases. Study further tries to locate the features with which various interventions can be enhanced. To explore the most recent findings, the present literature review looks into studies conducted in the period from 2010 to 2020.

Taking into consideration the objectives described above, the following research questions have been formulated:

#### Research Questions

1. Which interventions support various areas of SRL in its three phases in the context of online learning at higher education level?
2. Which technical features and representations of SRL support interventions, and which combinations of interventions are effective for SRL support?

## Methodology

**FIGURE 2:**
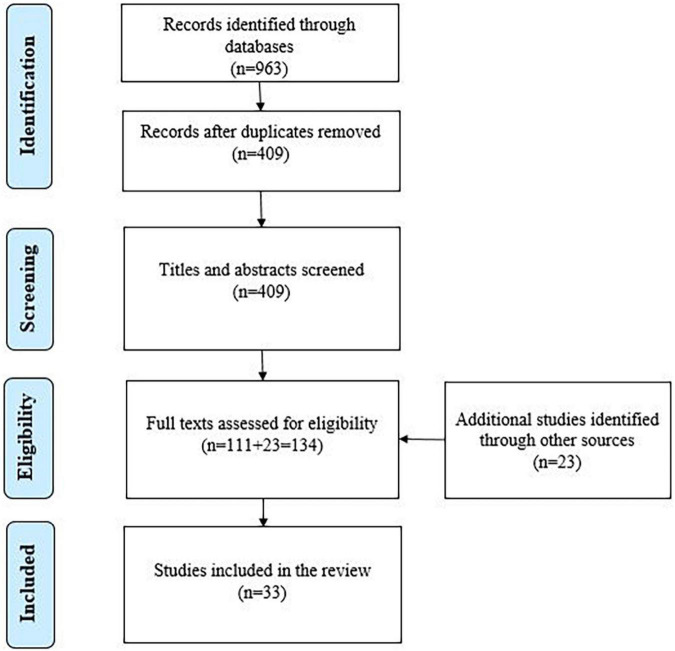
Selection process of the studies.

The present systematic literature review was conducted following the Preferred Reporting Items for Systematic Reviews and Meta-Analysis (PRISMA) approach (Moher et al., 2009). Consequently, the study selection process consisted of several phases: article identification, screening, checking for eligibility and final rigorous full text analysis (see Figure 2). The analysis was conducted according to the data analysis framework developed by the authors specifically for this study.

### Search and Selection Procedures

#### Search Terms Defined and Databases Selected

In an attempt to thoroughly cover all aspects of the research, first, the main concepts covered in the RQ were clearly identified. The table below presents the concepts along with their synonymous terms used under each concept category.

**TABLE 1:** Concepts explored and included in the search.

Concept 1	Concept 2	Concept 3
Supporting Scaffolding Facilitating	Self-regulated Self-control	Distance learning Web-based learning Online learning E-learning Digital learning

Based on the identified concepts and related terms, a relevant search phrase was created, which is presented below:

(“support*” OR scaffold*” OR “facilitat*”) AND (“self-regulat*” OR “self-control” OR “SR” OR “SRL” OR “self-regulated learning”) AND (“distance learn*” OR “web-based learn*” OR “online learn*” OR “e-learn*” OR “digital learn*”).

Since the aim of the search was to detect any potential SRL support mechanism, no search words such as tool, framework or intervention were specified. Rather, the word *Support/Scaffold*/*facilitate* was expected to naturally lead to the identification of the diverse possibilities of SRL support methods. Further, even though the concept of SRL can be further broken down into smaller constituent categories (cognition, metacognition, motivation, emotion), such elaboration was considered unnecessary based on the assumption that the term *self-regulated learning* would be sufficient for locating studies covering various constituents of SRL.

The search for studies focusing on the supporting intervention of SRL was conducted in the Web of Science and EBSCO, two of the biggest database platforms focusing intensively, among other areas, on the field of education. The platforms were expected to have the most relevant articles for our study purposes. Advanced search was used in both databases using the search formula presented above.

Since the focus of the study was to identify the interventions most up to date in nature, the search was restricted to the period from 2010 to 2020. The studies selected had to come from peer-reviewed journals, conference publications and dissertations. Another limitation applied during the search was with regard to language—only those articles published in English were targeted. Besides other obvious reasons, English is the common language for all authors involved in this study and facilitated the required validation processes involved in the study.

#### Study Selection Criteria

The following inclusion criteria were used for the study selection purposes at the abstract as well as full text screening level:

1. The study makes an explicit link to SRL and focuses on at least one area of SRL (cognition, metacognition, emotion, motivation).
2. The study is empirical in nature, and attempts to measure the effect of an intervention(s) on SRL.
3. The study focuses on distance learning (covering all types of digital media).
4. The study is conducted with students at higher education level.

#### Search Process

Searching Web of Science and EBSCO for relevant articles resulted in identifying 351 and 729 articles in each database, respectively. EBSCO database automatically eliminated the duplicates identified in its search result, leaving the total at 512 articles. EBSCO and Web of Science articles together amounted to a total of 963.

When both search result sets were exported to the web version of EndNote, an additional search for duplicates was performed, and 496 articles were eliminated automatically as well as additional 58 ones manually, leaving the total of merged database results at 409. As a result of further screening – browsing the titles and abstracts of the articles – 111 studies were found to be meeting the established inclusion criteria and selected for further eligibility check. In the process of full text screening, 23 additional relevant studies were identified through the references of the selected articles and added to the pool of 111. After the final full text analysis, 33 articles were selected as meeting the inclusion criteria. The reviewed articles include four that describe two or more studies, looking at differentiating effects of certain features and/or combination of features (Kauffman et al., 2011; Bannert and Mengelkamp, 2013; Lehmann et al., 2014; Wäschle et al., 2014) of an intervention. Thus, the total number of actual studies investigated amount to 38. Additionally, there are three studies in the review which focus on the same intervention, *MetaTutor*, each focusing on the effects of the given platform on SRL from a slightly different perspective. The multifunctional and explicitly SRL support directed nature of the given medium must be the reason of the recurring interest. More information about the articles can be found in Supplementary Appendix B. The whole systematic literature review process is captured in Figure 2.

#### Validity

Two authors examined the studies separately. The validation was conducted at title and abstract as well as full text level (30 articles validated at each level) according to the inclusion criteria outlined. Cohen’s Weighted Kappa test revealed the range of.82 to.92 reliability at the title and abstract, and from full agreement to.88, in the full text level.

### Data Analysis

A frame of analysis was created by the authors to facilitate systematic capturing of the information related to the studies included in the present literature review. The validation was ensured through the rounds of discussions and refinement of the frame, as well as actually applying it to 5 studies before its employment for wider scale study analysis (see Supplementary Appendix B). For validation purposes, the authors held a discussion with regard to the components included in the table and finalized its structure. Additionally, two authors analyzed 5 studies using the given table and compared the extracted data for consistency and validity checking purposes.

#### Key Term/Concept Definitions

In the context of this study, several terms are of high importance and recurring throughout the study. Firstly, the term *intervention* will be used to refer to all possible methods of SRL support, including the concrete digital tools as well as more general support types such as pedagogical frameworks, platform designs and quality factors explored for SRL support. Another key umbrella term used in the current study is *distance learning*, which refers to all types of learning not taking place in F2F format and implemented distantly with the help of technology, ranging from Massive Open Online Courses (MOOCs) and Learning Management Systems (LMS) to Hypermedia Learning Environments (HLE).

As for the key concept involved in the present review, *self-regulation*, it is defined, in general terms, as a process through which self-generated thoughts, emotions and actions are planned and adapted to reach the established goals (Zimmerman, 2000). As for SRL, according to Panadero (2017), it is an extraordinary umbrella under which a considerable number of variables that influence learning (e.g., self-efficacy, volition, cognitive strategies) are included within a comprehensive and holistic approach [.]. [It is a] core framework to understand the cognitive, motivational and emotional aspects of learning (p. 1).

In the present study, in accordance with the definition of Panadero (2017), SRL is perceived as a multi-component, cyclical process, involving several stages and areas (see also section “BACKGROUND”).

#### Study Component Categories Clarified

##### Framework of Analysis

Descriptive data about the articles includes the information about the study *domain*, which requires further categorization. Disciplines such as Biology, Chemistry and Geography are classified under *Science*; technology-related disciplines such as Computer Science and Programming under *Technology*; Research, Educational Sciences and Educational Psychology falls under *Education*; and *Medicine* is presented separately. As for the study medium, different ways of referring to it was adopted in various studies. Whereas in some studies learning environment was described in broader terms (e.g., LMS), in others the course type (e.g., MOOC) or the qualities of the systems working behind the platform were reported (e.g., Hypermedia). This made clear, broader categorization of the information about the learning media challenging. Thus, the analysis framework captures the information as it is presented in the studies reviewed.

With regard to the research quality related section of the table, the following categories were formed: *Longitudinal*, referring to the course-long and more extensive studies, and *Cross-sectional*, referring to shorter studies, conducted during one or more learning sessions.

Outside the analysis framework, the interventions were further categorized with terms *Micro* and *Macro*— *Micro*, focusing on helping learners at the task level, and *Macro*, facilitating SRL throughout the course and/or at the study cycle level, types, which are then further grouped according to the phase and area of SRL they support.

As for the more concrete categories related to the interventions, broadly, they fall into following categories (a). *Prompts*, which are largely defined as “recall and/or performance aids” (Bannert, 2009). They do not teach new information, rather the assumption is that students have previously mastered the concept/knowledge, and need help with learning execution. According to Bannert, they “are scaffolds to induce and stimulate students’ cognitive, metacognitive, motivational, volitional and/or cooperative activities during learning” (2009). Other larger categories of the reviewed interventions include (b) *various tools* (time logging, note taking, group awareness, assessment), which are embedded instruments facilitating the process of SRL in various ways. (c) *Instructional designs* built on SRL principles are other interventions providing broader, framework-based support, affecting the whole dynamics of the learning experience. (d) Other interventions include technical features/additions to the system/tool such as *visualization*, *social comparison*, as well as *video/text enhancement* functionalities. Learning environment related *quality factors* (system, service, information) are yet other components to consider while identifying SRL support mechanisms (e). For more clarity and better understanding of the interventions explored, descriptions of intervention related terminology is provided in Supplementary Appendix A as well as in the Results section below. As for the terminology used for referring to the *effects* of the interventions included in Tables 3–5, these have been extracted from the original articles and are common in the field needing no further interpretation or categorization.

#### Effect Size Calculation

To present comparable effects of the SRL interventions explored in the included studies, the effect size of these intervention on learners’ SRL skills (for concrete examples of dependent variables, see Tables 3–6) was calculated using standardized *Cohen’s d* (*d*). The effects were calculated based on the presented data in the particular studies: *t*-value, *f*-value, χ2-value or *p*-value of the tests along with sample sizes reported. In a few cases, where not enough analysis data was provided, calculation of the effect size was not possible.

## Results

### Descriptive Data

The studies included in the review come from peer-reviewed scientific journals, dominated by the journal Computers in Human Behavior (8), and several from the International Conference proceedings (3) and one dissertation. The study domains involved are mostly from the field of education (14), technology (12), science (7) and medicine (4); thus, the study topics/tasks as well are complex in nature and require intense support in distance learning formats. As for the learning media, they range from MOOCs and Learning Management Systems (LMS) to adaptive multi-agent hypermedia learning environments. Most of the studies are conducted in countries with highly developed educational technologies infrastructure (i.e., 24% in Germany, 24% in the United States).

As for the methods applied, as defined by the inclusion criteria, studies reviewed are empirical in nature. In vast majority of cases data collection tools and data analysis methods were largely reported. The data for analysis was collected through a combination of various sources such as log data, video protocols, observation protocols, archived forum discussions and various types of validated targeted questionnaires (e.g., Motivated Strategies for Learning Questionnaire (MSLQ) (Pintrich and de Groot, 1990); Perceived Stress Questionnaire (PSQ) (Levenstein et al., 1993) and procrastination questionnaires (Lay and Silverman, 1996), Intrinsic Motivation Inventory (IMI) and the PANAVA inventory (Schallberger, 2005). However, the level of detail and consistency when referring to certain areas/concepts involved varied across the studies. While the domain of the study, as well as participant number and age was almost always reported, there were some gaps with regard to the information about the validity check procedures of the data collection instrument(s). As for the study variables, for instance, the learner-related characteristics which might have a differentiating effect on the efficiency of certain SRL interventions in given contexts, were not consistently reported in the studies reviewed, and were only investigated in a few cases (Ifenthaler, 2012; Verpoorten et al., 2012; Bannert and Mengelkamp, 2013; Lehmann et al., 2014). Thus, due to the lack of enough data in this direction, we could not include this information in a systematic manner in our analysis framework.

The interventions identified focus both on micro (task/activity, e.g., Matrix note-taking tools; Kauffman et al., 2011) and macro (study cycle) level (e.g., Planning and Reflection Protocol; Wäschle et al., 2014). The micro level interventions tend to be the shorter term investigations, whereas macro level interventions are investigated in the context of the longer term designs (e.g., Alexiou and Paraskeva, 2015; Yeomans and Reich, 2018). See more in Supplementary Appendix B.

### Interventions Supporting Various Areas of Self-Regulated Learning in Distance Learning Environments at Higher Education Level

Table 2 summarizes the findings in a numerical format with regard to the SRL support interventions, which are categorized and presented according to the SRL phases (preparation, performance, appraisal) and areas (cognition, metacognition, motivation, emotion) they target and have an effect on. The subsequent sections further elaborate on the details of the interventions identified in the studies reviewed.

**TABLE 2:** Self-regulated learning (SRL) phases and areas targeted by the SRL support interventions^1^.

SRL Areas	SRL Phases			Total
	Preparation	Performance	Appraisal	
Cognition	0	13	0	13
Metacognition	9	15	5	29
Motivation	2	13	1	16
Emotion	0	6	0	6
Total	11	47	6	

*^1^ The total numbers presented in this table do not reflect the amount of each unique intervention but rather the multiple SRL areas and phases they target (in most of the cases an intervention affects more than one area of SRL and/or more than one phase).*

As can be observed from the table below, by far the biggest number of the studies explore interventions that support metacognition regulation, followed by motivation and cognition. The biggest gap detected concerns emotion regulation. As for SRL phases, the biggest emphasis can be seen with regard to the performance phase, whereas preparatory as well as appraisal phases are underexplored in all areas of SRL except for metacognition, where preparation phase support is also well covered.

#### Interventions Supporting Cognition Regulation

Prompts have been identified as among the most widely researched interventions to support cognitive (as well as metacognitive) areas of SRL. *Adaptive Content and Process Scaffolds* involving human tutor (Azevedo et al., 2011) have been proven to be an effective SRL support intervention. Process-embedded, adaptive scaffolding, delivered by course assigned tutors in real time, in a personalized manner, has a positive impact on learners’ cognitive (content evaluation, note-taking, hypothesizing, re-reading) as well as metacognitive (planning, monitoring progress, reflection, goal directed search, help-seeking) strategy use. However, at the same time, the study reveals the fact that even though extra help provided with regard to the content, in addition to the process support, is conducive to declarative knowledge gain and has a positive effect on cognition regulation, too much support (content as well as process) might result in overdependence, and hence, lesser transferable SRL skills. See Table 3 below.

**TABLE 3:** Cognition regulation support intervention and the areas affected.

SRL Phases	Intervention^1^	Strategy/Area Affected	Effect^2^
**Preparation**	N/A	N/A	N/A
**Performance**	Tutor provided adaptive Content and Process Scaffolds (5)	Reading/note-taking Re-reading Hypothesizing	*d* = 0.71, CI[0.34, 1.0] *d* = 0.71, CI[0.33, 1.0] *d* = 0.70, CI[0.33, 1.0]
	Pedagogical agent scaffolding (instructional prompts and feedback) (9)	Note-taking, summarizing, content evaluation	*d* = 0.51, CI[0.07, 0.94]
	Matrix and outline note-taking tools (11a)	Accurate and relevant information localization	*d* = 2.4, CI[1.3, 3.5]
	Note-taking tools combined with self- monitoring prompts (11b)	Note-taking (more notes taken) *monitoring component - for conventional note-taking	*d* = *0.96*, CI[0.56, 1.3]
	Peer-peer formative feedback in asynchronous fora, stimulated and monitored by tutor (12)	Sharing and comparing content understanding, Meaning construction	N/A
	Generative learning strategy prompts and metacognitive feedback (15)	Highlighting main information, note-taking, refining knowledge by revisiting materials	*d* = 0.26, CI[0.02, 0.52]
	Personalized e-journals + self-reflection prompts (18)	Elaboration, rehearsal	*d* = 0.89, CI[0.33, 1.4]
	The negative impact of media diversity (21)	Increased cognitive load	*d* = −0.32, CI[−0.40, 0.24]
	Learning Framework *(SR-INSPIRE us)* with adaptive presentation support technique (25)	Improved cognitive processing and cognitive achievement	*d* = 0.71, CI[0.00, 1.4]
	Group awareness tool (26)	Information processing Selection of main ideas	*d* = 0.96, CI[0.54–1.38] *d* = 0.86, CI[0.44–1.27]
	Prompts on help-seeking (28)	Help-seeking activity about relevant content	*d* = 0.26, CI[0.01–0.52]
	Instructional design workflow – PBL and SRL combined (32)	Rehearsal, elaboration, organization	*d* = *0.60*, CI[0.12–1.0] *d* = 0.81, CI[0.32-1.2] *d* = 0.70, CI[0.22-1.1]
	Planning and reflection protocol (1)	Rehearsal Organization, elaboration	*d* = 0.59, CI[0.01–1.1]
**Appraisal**	N/A	N/A	N/A

*^1^ The numbers in this and subsequent Tables 2-4 refer to the studies in Supplementary Appendix B.*

*^2^ 95% Confidence Interval (CI) is set for the effect size.*

Another study by Duffy and Azevedo (2015), investigating the online learning platform *MetaTutor*, focuses on adaptive prompting and subsequent feedback provision coming from its platform-embedded pedagogic agents covering different areas of SRL strategy support. Agents prompt participants to deploy specific SRL strategies— goals and sub-goal setting and staying mindful of their overall learning goal. The learners are also prompted to activate their prior knowledge, write summaries, assess the relevance of the content, take notes, assess their understanding and re-read sections of the text. The agents then give feedback regarding the accuracy and relevance of the practices employed, which has a positive impact on cognition regulation strategy improvement such as note-taking, summarizing and inferring skills as well as content evaluation ability (for metacognition and motivation related effects, see below in sections “Interventions Supporting Metacognition Regulation” and “Interventions Supporting Motivation Regulation”). Since the study also investigated the effect of the learner goal orientation, as a factor, it revealed that such support is more useful for performance- rather than mastery-oriented learners, the authors speculating that intensive prompting and feedback provision might not be creating a challenging enough learning experience for mastery-oriented students. *The Planning and Reflection Protocol*, embedded in an LMS, which primarily focuses on metacognition regulation support, helping learners in the process of planning, goal setting and reflection, proved to have a positive effect on cognition regulation strategy use as well. Learners, by setting specific goals and then reflecting on what has been learned and what needs further adaptation, manage to engage in more targeted and conceptual learning (Wäschle et al., 2014).

The interventions which enhance learner cognitive regulation (as well as have an effect on metacognition, motivation and emotion regulation, see Sections “Interventions Supporting Metacognition Regulation,” “Interventions Supporting Motivation Regulation,” and “Interventions Supporting Emotion Regulation” below) through group awareness mechanisms applied in collaborative contexts include *pie chart* reflecting learners’ posting for behavioral awareness and *group interaction diagram* for social awareness as well as *cloud tags* capturing the main concepts coming up in the process of collaboration (Ma et al., 2020). Application of these interventions resulted in better information processing by learners as well as more accurate identification of and focus on the relevant information in the process of learning. The benefits of collaborative practice were further demonstrated in other studies. Receiving and offering constructive peer feedback in discussion forums helps learners build a new understanding and perspectives, leading to the construction of new knowledge (Gikandia and Morrowa, 2016). Collaboration also helps with elaboration and rehearsal strategy improvement, the practice which is especially helpful in less structured contexts such as problem-based learning (Paraskeva et al., 2017).

Further, at the task level, the interactive nature of the tools used (available functionalities such as note-taking, text highlighting, annotation and summarizing), which allow learners to get actively engaged in the learning process rather than remain in a passive recipient’s role, improves their cognitive regulation strategies. The *Video Mapper* and *MetaTutor’s* reading platforms offer such environments (Lee et al., 2010; Delen et al., 2014). Note-taking tools, especially the multi-dimensional ones (Matrix and Outline), which contribute to putting learners in charge of collecting the right information for learning and processing, have also been found helpful with cognition regulation. While taking notes, learners engage in prioritizing and trying to discern what is essential and what is secondary level information. Capturing the main concepts in such a structured manner makes information processing easier, allows more focused revision and elaboration (Kauffman et al., 2011) and, ultimately, is conducive to improved learning and information retention.

Another important factor to be considered while trying to help learners with cognitive processing and avoidance of negative overload is to carefully plan the learning environment using well-structured modes of information presentation as well as efficient use of diverse media formats. Even though there is evidence pointing to the positive effects of multimedia use in the instructional process on learners’ cognitive processing and increased level of learning, overall, inefficient application of the diverse media can have reverse effects. Using multimedia for delivering the content/messages irrelevant to learning and/or providing redundant/overlapping information through various forms of media (e.g., text, visuals, audio, graphical) leads to diverting learners’ attention from the essential to non-essential information processing. This results in extraneous cognitive workload and confusion on learners’ part, which further hinders self-regulation and is conducive to lower levels of knowledge acquisition (Mayer et al., 2001; Lange and Costley, 2019).

#### Interventions Supporting Metacognition Regulation

Information about metacognition regulation support interventions is captured in Table 4 and further elaborated in the text that follows to provide further details.

**TABLE 4:** Metacognition regulation support interventions and areas affected.

SRL Phases	Metacognition	Strategy/Area Affected	Effect^1^
**Preparation**	Planning and reflection protocol (1)	Reduced procrastination Improved goal specificity	*d* = 0.62, CI[0.04, 1.2] *d* = 0.68, CI[0.10, 1.2]
	Metacognitive prompts (3b)	Orientation, planning and goal setting	*d* = 0.84, CI[0.19, 1.4] *d* = 0.86, CI[0.21, 1.5]
	Metacognitive prompts + training in their use (3c)	Planning Goal specification	*d* = 0.58, CI[0.04, 1.2] *d* = 1.0, CI[0.48, 1.8]
	Fading/adaptive prompts (4)	Content-goal relevance and existing knowledge evaluation	*d* = 0.50, CI[0.18, 0.81]
	Tutor provided adaptive Content and Process scaffolds (5)	Planning-prior knowledge activation, setting sub goal	*d* = 1.0, CI[0.63, 1.4]
	Pedag. agent provided instructional prompts and feedback (9)	Planning and prior knowledge activation	*d* = 0.50, CI[0.07, 0.94]
	E-portfolio based on SRL framework (16)	Planning	*d* = 0.84, CI[0.20, 1.4]
	Instructor and institutional support and Course quality (19)	Independent goal setting, planning	*d* = 0.34, CI[0.18, 0.50] *d* = 0.30, CI[0.15, 0.46]
	Pedagogical agent-supported monitoring/reflection prompts (23)	Goal setting, planning	*d* = 1.9, CI[1.5, 2.4]
**Performance**	Metacognitive prompts (3b) + training in their use (3c)	Monitoring Search and judge	*d* = 0.92, CI[0.27, 1.5] *d* = 0.69, CI[0.06, 1.3]
	Fading/adaptive prompts (4)	Management of progress toward goal	*d* = 0.50, CI[0.18,0.81]
	Tutor provided adaptive content and process scaffolds (5)	Time and effort planning Goal directed search	*d* = 1.0, CI[0.63, 1.4] *d* = 0.61, CI[0.24, 0.98]
	Automated adaptive time management enabling system (6)	Less procrastination and cramming	*d* = 1.2, CI[0.68, 1.8]
	Time logging tool (7)	Time management and planning	*d* = 0.65, CI[0.09, 1.2] *d* = 0.50 CI[0.04, 1.0]
	Pedag. agent provided instructional prompts and feedback (9) Fading effect (4)	Help-seeking, content evaluation, judgments of learning Progress toward goal	*d* = 0.51, CI[0.07, 0.94] *d* = 0.50, CI[0.18, 0.81]
	Radar visualization (10)	Starting Earliness of submission	*d* = 0.43, CI[0.01, 0.81] *d* = 0.24, CI[0.15, 0.64]
	Visualized feedback with social comparison (13)	Timeliness (reduced procrastination)	N/A
	E-portfolio based on SRL framework (16)	Time management Monitoring	*d* = *2.1*, CI[1.3, 2.9] *d* = *2.8*, CI[2.0, 3.7]
	Pedagogical agent-supported monitoring/reflection prompts (23)	Performance stage skills Self-observation	*d* = *1.7*, CI[1.2, 2.1]
	Group awareness tool (26)	Time management, self-testing, study aids	*d* = 1.2, CI[0.77, 1.6] *d* = 1.1, CI[0.74, 1.6] *d* = 0.98, CI[0.56, 1.4]
	Self-assessment scripts (30)	Learning strategies	*d* = *0.55*, CI[0.07, 1.0]
	Instructional design workflow – PBL and SRL combined (32)	Metacognition Help-seeking	*d* = 0.44, CI[0.03, 0.91] *d* = 0.42, CI[0.05, 0.89]
	Self-directed metacognitive prompts (31)	Goal orientation (visiting and spending time on relevant pages; non-linear navigation) *More transferable skills*	*d* = 0.65, CI[0.17, 1.1] *d* = 0.58, CI[0.10, 1.0] *d* = 0.63, CI[0.15, 1.1] *d* = 0.44, CI[03, 0.91]
**Appraisal**	Metacognitive prompts (3b) + training in their use (3c)	Evaluation	*d* = *0.79*, CI[0.15, 1.4] *d* = 0.57, CI[−0.57, 1.2]
	Fading/adaptive prompts (4)	Knowledge evaluation	*d* = 0.96 CI[0.52, 1.4]
	Peer feedback in asynch. forum (12)	Self-assessment	N/A
	Pedagogical agent-supported monitoring/reflection prompts (23)	Self-reflection	*d* = 2.4, CI[1.93, 2.96]
	E-portfolio with techno-pedagogic design (29)	Deeper reflection and revisiting learning evidence	N/A

*^1^ For the studies marked as N/A not enough data was available to calculate the effect size.*

As in the case of cognition, with regard to metacognition as well, prompts have been found to be among the most prominent interventions supporting metacognition regulation. The findings with regard to prompts indicate that specific prompts (e.g., pop-up prompts at various SRL phases reminding learners of using relevant SRL strategies) are significantly more efficient and conducive to more positive effects on metacognition regulation, especially when combined with feedback (Duffy and Azevedo, 2015), than prompts that are generic in nature (Bannert and Mengelkamp, 2013). The importance of carefully designing prompts and attributing them context specific nature is especially pronounced in less structured learning contexts, and with less experienced learners (Verpoorten et al., 2012).

Further, training with regard to understanding and following the prompts has also been found helpful. However, the positive effect of such explicit training can be observed only with less experienced and less motivated learners, whereas almost a reverse influence can be observed on more advanced learners with high intrinsic motivation (Bannert and Mengelkamp, 2013). Similar findings were also reported in the study by Duffy and Azevedo (2015) with regard to over supporting learners with high mastery orientation (intrinsic motivation). The authors speculate that it might be the lack of challenge inherent in the scaffolding that undermines mastery-approach learners’ interest and makes them overwhelmed rather than motivated. Despite being conducive to better knowledge gain and better metacognition regulation strategy use (planning, monitoring progress, reflection, goal directed search, help-seeking), tutor-provided content and process directed support can result in learners’ overdependence on external help and hence, lesser transferable SRL skill development (Azevedo et al., 2011).

*Adaptability* and *self-directed* nature are other features that have been found to make prompts more effective. An adaptable, initially more frequent but progressively fading prompting contributes to the increase of SRL strategy deployment (Bouchet et al., 2016). Another way identified to support the metacognition regulation process is through *self-directed prompting*, which involves learners themselves in the process of configuring their own prompts by selecting relevant SRL strategies from a template and determining the time stamps for the prompts to pop up and support them in the process of learning. Such self-directed metacognitive prompts have been found to help learners engage in platform navigation in a more targeted rather than linear manner—learners identified and visited more relevant pages/materials and, overall, spent more time on learning. Further, both adaptive and self-directed prompting have also been found to have a transferable effect, manifested through the fact that at a later stage, in the context of reduced or no metacognitive prompting, learner-initiated regulatory activities still increased (Bannert et al., 2015).

The positive effect of the comprehensive and personified approach adopted with regard to prompting was witnessed in the study by Yılmaz et al. (2017). During the learning process, questions were directed to the learners by an animated and audible video-based *Pedagogical Agent* (PA) at macro SRL-phase level, encouraging planning, monitoring and reflection (at first, prior knowledge prompts are asked, expectations are set with regard to the course content; then, self-monitoring prompting takes place, and at the end of the week, reflection happens), as well as at micro, learning material level (prompting about how well the videos and/or texts have been understood). The intervention was found effective for metacognitive strategy improvement across all SRL phases. However, the biggest effect was observed with regard to the performance phase.

Interventions specifically focusing on and positively influencing time management strategies include systems that help learners stay conscious of their time spent on learning and avoiding cramming and procrastination. In this regard, the *Automated Adaptive Time Management Enabling System* (Khiat, 2019) with special features such as visual reinforcement (e.g., visual representation of the study plan on the main page), adaptive release of study materials, learning monitor and learning motivator messages was found helpful. The personalized nature of the notifications sent via a mobile linked application system and the optimal timing of the sent reminders, together with the social comparison feature enabling learners to analyze their own progress against their peers and teacher-set expectations, have also been shown to be helpful for learners to better organize their time while learning (Tabuenca et al., 2015; Jivet, 2016; Ma et al., 2020).

There are interventions that focus on SRL and particularly on metacognition regulation support in a more comprehensive manner, such as portfolios and platforms with specific SRL instructional workflow design. E-portfolio α*pot2iMySelf*, for instance, requires learners to reflect on their SRL skill use throughout all three learning phases and complete the portfolio with relevant information throughout the course, which helps learners with consistent and systematic planning and monitoring of their progress (Alexiou and Paraskeva, 2015). E-portfolio *Transfolio* with techno-pedagogical designs, coupled with teacher led procedural guidance as well as the need on the learners’ part to dialog with the teacher and provide learning evidence was also found to be conducive to learners’ increased reflection, self-assessment and learning adaptation strategies (Torras and Mayordomo, 2011). Additionally, an online platform with comprehensive *SRL instructional workflow design* (*apT2CLE*), founded on PBL collaborative model, and having instructor support available as needed, was also found to be supportive to learners through the learning process with their metacognition regulation strategies (Paraskeva et al., 2017). Pedagogic design as well as the overall quality of the learning system, instructor provided support and intuitive course structure helps learners to better self-regulate in an online learning environment (Albelbisi and Yusop, 2019).

The benefits of instructor supervision of the learning process in distance learning environments as well as the self-regulatory power of cooperative learning contexts have been demonstrated by Gikandia and Morrowa (2016). Specifically, learners’ active participation and collaboration within (a) the topical asynchronous discussion forum, (b) open forum to share developing thinking and work in progress and (c) forum for students to share their polished artifacts and receive *peer formative feedback*, while the whole process is being stimulated and monitored by the teacher, was found to be conducive to learners engaging in more targeted goal setting, intensive reflection, self-monitoring and self-assessment.

The potential of assessment instruments such as assessment scripts and rubrics has been investigated and shown to be helpful for SRL (Panadero et al., 2013). Namely, while the scripts were helpful with more complex tasks and deep learning—better goal setting, deeper reflection and self-assessment— the rubrics were useful for staying focused on the learning process, monitoring and meeting the set expectations (specified in the rubrics) in the context of low to medium complexity tasks.

#### Interventions Supporting Motivation Regulation

As the results of the current literature review show, the motivation regulation support has been largely associated with clarity with regard to learning objectives, learner autonomy, collaboration, the opportunities to analyze and compare one’s own learning to standard performance and the quality of the learning environment. The information about motivation regulation support interventions is captured in Table 5 and further elaborated in the text that follows.

**TABLE 5:** Motivation regulation support interventions and areas affected.

Phases	Intervention	Strategy/Area Affected	Effect
**Preparation**	Directed pre-flection prompts (2b)	Positive activation through step-by-step guidance	*d* = 0.39, CI[0.09, 0.87]
	Planning and reflection protocol(1)	Self-efficacy	*d* = *0.63*, CI[0.06, 1.2]
**Performance**	Mastery grids *with social comparison* (8)	Engagement and effort allocation	*d* = 1.05, CI[0.61, 1.4]
	Pedag. agent provided instructional prompts and feedback (9)	Engagement (time viewing materials)	*d* = 1.1, CI[0.72, 1.4]
	Peer feedback in asynch. topical fora, stimulated and monitored by tutor (12)	Increased engagement/interaction Self-value	N/A
	Visualized feedback with social comparison (13)	More access to videos, attempts at *graded* quiz questions, forum visits	N/A
	Pre-planning prompts (17)	Greater persistence and completion	*d* = 0.61, CI[0.08, 1.1]
	Online platform with learner-style oriented instructional design (14)	More time spent on materials	*d* = 0.34, CI[0.32, 1.0]
	Enhanced video tool (20)	More engaged: spent more time on video material	*d* = 2.5, CI[1.9, 3.2]
	Group awareness tool in collaborative environment (24)	Increased number of contributions and interaction with peers	*d* = *0.49*, CI[0.05, 0.93] *d* = 0.76, CI[0.31, 1.2]
	Learning framework based on learner preferences (25)	Increased self-efficacy for learning and performance	*d* = 0.71, CI[−0.01, 1.4]
	Group awareness (visualized feedback) (26)	Self-efficacy More time learning Attentive learning	*d* = 0.52, CI[0.21, 0.83] *d* = 1.9, CI[1.5, 2.3] *d* = 2.5, CI[2.0, 2.9]
	E-learning WEB 2.0 (System, inform., service quality) (27)	Interaction/cooperation increase (with peers and content)	*d* = 0.37, CI[0.12, 0.62]
	Prompts on help-seeking (28)	More active participation Initiating discussions	*d* = *0.64*, CI[0.03, 1.2]
**Appraisal**	Automated adaptive time management enabling system (6)	Improved completion rate (persistence)	*d* = 0.58, CI[0.01, 1.1]

Engaging learners in setting their learning goals and planning their study process has been proven to have a positive effect on learners’ self-efficacy (Wäschle et al., 2014). Assessment rubrics and scripts also help with managing learning expectations and lay out the path toward achieving the goals. Such clarity with regard to the upcoming learning experience and set expectations positively impacts learner motivation—they become more engaged in the learning process due to reduced stress related to the complex tasks and have also been found to avoid difficult tasks they encounter in the process of learning less. However, increased self-efficacy and the feeling of contentedness have not been witnessed with regard to self-assessment rubrics and scripts, authors speculating that this can be explained by the absence of the feedback involved in the process, which would likely make the learning experience more fulfilling (P). Pre-planning prompts, which encourage learners to make learning plans at the beginning of the learning process and which then stay visible for learners’ further reference, have also been identified as having a positive effect on learners’ subsequent learning experience and persistence during the process (Yeomans and Reich, 2018). By being better prepared from the very outset, learners feel more empowered and choose not to “surrender” (persistence) in the face of potential challenges. Hence, the provided support assumes a predictive (and thus, preventive) nature and helps learners elaborate implementation strategies for achieving the set objectives while the intention is still strong. Another feature of such prompts that makes them effective is their targeted nature, which contributes to learners’ positive activation (Lehmann et al., 2014). The intervention was found to be particularly useful for novice learners in open learning environments such as MOOCs. The effect of the pre-planning prompts is further enhanced.

The benefits of systematic planning implemented through *weekly e-journals* and further supported by *reflective prompts* have been demonstrated by Fung et al. (2019). Careful planning and then reflection on the challenges encountered during the week and analyzing the methods applied/not applied to overcome those difficulties were found to be conducive to learners spending more time studying as well as making more effort during the online learning process. Such persistence and increased motivation is especially important for learners engaged in longer term courses.

Systematic reminders about the progress made, and explicit encouragement to make more effort helps learners stay mobilized and motivated. An Automated Adaptive Time Management Enabling System (also discussed in section “Interventions Supporting Metacognition Regulation”) sending learning monitors (reminder emails about progress) and learning motivators (personalized emails sent out to learners to compliment them on their achievements and/or encourage learners who are falling behind to do better) was found to be conducive to learners spending more time on material and more students completing the course successfully (Khiat, 2019). Similarly, explicitly reminding learners of the possibility and the need to ask for help in the process of learning by placing the prompts along learners’ individual workspace proved to be an encouraging factor for students’ increased participation and involvement in the study process (Schworm and Gruber, 2012).

Adaptability achieved through Open Learner Modeling (OLM) and its benefits have been demonstrated in several studies reviewed. In the *Mastery Grids* system, an intelligent interface for online learning content that combines Open Learner Modeling (OLM), adaptive navigation support and a navigation-oriented social comparison feature, learners can click on any topic cell of the interface and access diverse web-based “smart” practice content. The system can then process learner activity, estimate progress and incorporate feedback based on the log data. The adaptable and interactive nature of the system, possibility of receiving individualized feedback in a visual format and the comparison feature have proven to have a positive effect on learner engagement and overall efficiency (Guerra et al., 2016). Similarly, Learning Tracker (Jivet, 2016), using the low-level data from learner trace logs and condensing those into indicators, provides learners with individualized feedback on their performance through the spider chart visualization and allows social comparison. Such individualized and visual nature of feedback has proven to have a positive effect on learners’ persistence, translating into increased time spent on completing the quizzes as well as higher course completion rates. The study also revealed a longer term as well as a transferable effect (certain SRL aspects that were not explicitly targeted by the provided feedback, still improved over time) of the given intervention. The explanation could be, as the author suggests, interconnectedness of the learning activities involved in the SRL which cannot be completed independent of one another, and thus, the intervention acquiring a holistic effect on SRL.

An online collaboration environment with *Group Awareness (GA*) functionality, in a somewhat similar way to *Learning Tracker*, has been found helpful for boosting learners’ motivation regulation (Lin and Tsai, 2016). The intervention stimulates higher levels of peer-to-peer interaction and contribution to the learning process while allowing learners to observe group activity in the process of cooperative learning through their visualized log data (number of personal contributions made; feedback/evaluation provided; replies written; “likes” given in the process of cooperative learning). To remind students of using GA information, the given function is automatically displayed whenever students log in the system and is available upon demand. The motivational effect of GA has been found to be stronger and more sustainable with learners with higher self-regulation skills. Another intervention providing group awareness functionality was explored by Ma et al. (2020), which, alongside cognition and metacognition regulation (see sections “Interventions Supporting Cognition Regulation” and “Interventions Supporting Metacognition Regulation”), was also confirmed to be helpful with motivation regulation—the data indicates that the visualized feedback about one’s own as well as other students’ collaborative activities, provided to learners in a timely manner, can encourage students to work harder. The benefit of collaborative learning format on motivation regulation has also been proven by yet another study (Gikandia and Morrowa, 2016): namely, peer-to-peer interaction as part of the collaborative learning experience and the formative feedback, delivered in an asynchronous forum under the instructor stimulated discussion session, have been found to further stimulate learner engagement in the learning process.

Higher engagement in an online learning can also be induced by delivering learning materials through formats/tools which are interactive and allow diverse means of information processing. *MetaTutor* and *Video Viewer* tools, identified in the present study, offer such interactive learning opportunities. *Video Viewer*, for instance, allows interactive note-taking, viewing of supplemental resources, bookmarking and comprehension check opportunities during and after the viewing process, followed up with immediate feedback, which helps students to monitor and evaluate their learning progress (Delen et al., 2014). Similarly, an interactive reading tool (see also sections “Interventions Supporting Cognition Regulation” and “Interventions Supporting Metacognition Regulation” above) with text summarizing, annotating, bookmarking and highlighting, alongside cognition regulation strategy improvement, contributes to increasing learner engagement in the learning process and the sense of autonomy (Duffy and Azevedo, 2015).

Learning platforms having learner-directed, adaptive and individualized nature have been proven to have a positive effect on learners’ intrinsic and extrinsic motivation. For instance, *SR-INSPIRE us* is a *Learning Framework* supporting learners’ motivation (and emotion) regulation through a learner style-based, individualized approach. It aims at enabling learners to define and manage their learning path by means of providing a set of generic strategies and customized learning activities based on their learning preferences throughout the three phases of SRL (Souki et al., 2015). Learners are offered individualized content by changing the sequencing of the modules included in each content page (adaptive presentation support technique). Similarly, platforms allowing diverse modes of presentation of materials (watching, discussing, conceptualizing, trying out) and giving learners the choice to select the modes of instruction and materials of their preference, and each mode providing extra help for additional skill development specific to that mode (e.g., note-taking, for watching mode), motivated learners to spend more time on learning (Lee et al., 2016).

Other factors more general in nature have also been found to have a positive impact on motivation regulation— *system/tutor provided support* as well as *quality of the course* (design, appropriateness of outputs and ease of understanding of course materials) (see also section “Interventions Supporting Cognition Regulation” above). Especially with novice learners, with less developed technology skills, such factors determine the level of learner engagement and the anxiety level in the study process. Interestingly, factors such as information quality and service quality did not show any significant impact on learner SRL strategies in the same study (Albelbisi and Yusop, 2019), which can be explained by the fact that if the overall system (platform) quality and course design is not good enough, learners cannot even get to the stage of properly processing the information offered.

#### Interventions Supporting Emotion Regulation

The fewest interventions have been identified with a proven effect on emotion regulation in the present study. Information about the emotion regulation interventions is presented in Table 6 below.

**TABLE 6:** Emotion regulation support interventions and areas affected.

Phases	Intervention	Strategy/Area Affected	Effect
**Preparation**	N/A	N/A	N/A
**Performance**	Planning and reflection protocol (1)	Lower stress level (related to reduced procrastination)	*d* = 0.63, CI[0.06, 1.2]
	Learner style directed online platform (14)	More positive learning experience Controllability	*d* = 0.48, CI[0.18, 1.1] *d* = 0.76, CI[0.07, 1.4]
	Learner preference directed online platform (25)	Control of learning beliefs Less test-related anxiety	*d* = 42, CI[0.16, 1.0]
	Group awareness tool (26)	Anxiety control and reduced sense of loneliness	*d* = 0.52, CI[0.21, 0.83]
	Instructor and institution support Course quality (19)	Confidence, enjoyment, interest	*d* = 0.56, CI[0.40, 0.72]
	System, information, service quality) (27)	User satisfaction	*d* = 0.39, CI[0.13, 0.64]
	Assessment rubrics (30)	Reduced task anxiety/avoidance	*d* = 0.57, CI[09, 1.0]
**Appraisal**	N/A	N/A	N/A

As revealed by the present study, emotion regulation is in most cases closely related to motivation regulation, and similar interventions support these two SRL areas. As in the case of motivation regulation, collaborative and interactive learning environments, with open social comparison functionalities allowing learning about peers’ cognitive, behavioral and social activity patterns, result in reduced anxiety and boosted self-esteem. Cooperative activities also contribute to decreasing the feeling of loneliness and increasing the sense of relatedness and belonging to a learning community (Ma et al., 2020).

As in the case of motivation regulation, easy-to-navigate and well-designed course structure, together with instructor and system provided explicit support, as well as a simple interface have been shown to be conducive to less anxiety and emotional overload (Albelbisi and Yusop, 2019; Lange and Costley, 2019). Learner-directed online environments, allowing learners more autonomy and flexibility in the process of learning through personalized (learning style- and preference-oriented), adaptive modes of instruction have been proven to have a positive effect on emotion regulation. Learners in such environments have more control over their learning beliefs, higher self-efficacy, and consequently experience lower levels of test-related anxiety (Souki et al., 2015; Lee et al., 2016).

Additionally, interventions such as *assessment rubrics* that support learners to better prepare and orient themselves for the upcoming learning process help reduce negative emotions and task avoidance practice (Panadero et al., 2012). Likewise, lowered stress levels and reduced confusion were witnessed as a result of *Planning and Reflection Protocol* application (see also in section “Interventions Supporting Metacognition Regulation” above), which encourages learners to plan and set their learning goals before engaging in the learning practice, which results in better implementation of the learning process and less procrastination related anxiety (Wäschle et al., 2014).

### Technical Features, Representations of Interventions and Combination of Those Effective in Supporting Self-Regulated Learning

#### Identified Effective Technical Features of Self-Regulated Learning Support Interventions

*Open Learner Model* allows more individualization and adaptation of the online learning experience by tracing learners’ activities and making them available for analysis to the interested parties (i.e., teachers, learners). To make the raw data more easily digestible, v*isualization* comes into play, which has been proven to have a positive effect on learners’ metacognition as well as motivation regulation (Jivet, 2016)^1^. With the visualization, the type of visual being selected also becomes important. Since SRL is a multifaceted, multidimensional process, visualizations allowing multi-dimensional and multi-layered representation, such as radar graphs, line charts, heat maps, mastery grids, cloud tags and interaction diagrams come into play (Wäschle et al., 2014; Guerra et al., 2016; Jivet, 2016; Ilves et al., 2018; Ma et al., 2020), with the intentional use of different colors to denote different aspects and quality of learning (Wäschle et al., 2014; Guerra et al., 2016). Ilves et al. (2018) tested the effect of *radar* versus *textual feedback* on performance and mastery-oriented students’ SRL skills (starting the learning process and earliness) and found a positive effect of radar visualization over the textual one (*d* = 0.43, CI[0.01, 0.81]) as well as the advantage of the textual visualization over no visualization option (in case of performance-oriented learners—*d* = 0.1.5, CI[1.1, 2.0] and mastery-oriented students –*d* = 0.42, CI[0.08, 0.77]). Interestingly, textual visualization did not have a favorable effect on scheduling, i.e., dividing the work across multiple days—the visualizations did not increase the number of days during which the students worked on the assignments, the difference between the groups being statistically significant (*p* = 0.031). The authors speculate that the possible explanation could be that the performance-oriented learners may have tried to gain all the exercise points as fast as possible, ignoring the feedback related to spacing out their effort over a longer period of time. Additionally, when it comes to academic performance, the highest performing students, regardless of the visualization, earned the highest scores, giving grounds to speculate that students who have strong task related or self-regulatory skills do not benefit from the external feedback provided by the visualizations as much as students with weaker skills (Ilves et al., 2018).

The *social comparison* feature, which allows analysis of students’ performance against standard expectation/class average/previous successful learners, was also explored in combination with the learner log data based visualization function, and was found beneficial for learners’ motivation, metacognition as well as cognition skills (Guerra et al., 2016; Jivet, 2016; Ma et al., 2020; see also studies N13, 26, 8, 13 in Tables 3–6 above). Further, the study by Ilves et al. (2018) described above investigated the effect of the *comparison feature* administered through layered radar graphs (student’s performance displayed in a blue layer and the average performance of all students in the course in a gray layer) and found that while beneficial for all types of learners, visualization without such comparison function might even have a reverse impact on performance-oriented students, who draw their motivation from outperforming others (*d* = −0.26, CI[−0.68, 0.14]. With regard to the social comparison feature, it has to be further observed that alongside its positive effect, it might also have a somewhat restricting influence on the diversity of student navigation, resulting in learners mimicking each other’s behavior and following unified learning patterns. To address the given downside of this feature, authors suggest combining it with personalized recommendation technologies (Guerra et al., 2016; Jivet, 2016).

#### Combination of Interventions for an Enhanced Self-Regulated Learning Support Effect

Some studies explicitly emphasize the necessity of combining several interventions in order to have a significant effect on SRL. Examples identified in the present study include *planning e-journals* combined with *self-reflection prompts* closely mapped with curriculum activities and assessment (Fung et al., 2019). *Note-taking tools* (Matrix, outline and conventional) when combined with *self-monitoring prompts* that encourage students to review their notes before moving on to the next activity have proven to have an enhanced effect on learners’ cognitive strategy use (more rehearsal and deeper analysis of the taken notes) as well as self-monitoring efficiency. Such combination is especially helpful with least supported (conventional note-taking) learners and more observable in the case of more complex tasks (*d* = 0.96, CI[0.56, 1.3]), (Kauffman et al., 2011), a fact that might indicate that more elaborate interventions on their part have more enhanced effects when combined with further scaffolding tools. Yet another study (Lee et al., 2010) showed the effectiveness of the task-based *generative learning strategy prompts* in combination with *the monitoring feedback* only (*d* = 0.26, CI[0.02, 0.52]). The generic nature of prompting, which, used on its own, was found not to have a significant effect (Bannert and Mengelkamp, 2013), seems to be boosted with more details and individualization coming in the form of monitoring feedback.

In a qualitative study by Gikandia and Morrowa (2016), the effect of *peer-to-peer feedback* in collaborative online learning contexts was shown to be enhanced with the detailed *assessment guidelines and analytical rubrics*. Such rubrics play a key role in supporting students to monitor their peers’ progress, and provide valuable feedback. The process further benefits from tutor supervision.

The important role of teacher involvement has also been proven with regard to the SRL interventions which are more complex in nature. SRL e-portfolio is a multifunctional and multi-component tool, the functionality and use of which need to be properly understood in order to reach the intended effect (Torras and Mayordomo, 2011). The preliminary preparation of students for the efficient use of the intervention has also been confirmed by another study on metacognitive prompting by Bannert and Mengelkamp (2013), which focuses on combining the administration of prompts with training on their use.

## Discussion

Operating efficiently in online learning environments is not an inherent competence that higher education students possess. Rather, it is a skill that needs to be developed in the process of learning and requires explicit support and training at an initial stage. The less experienced the learner and the more conceptually rich the learning domain, the more such help is needed. The present study investigated the SRL interventions that were found helpful for supporting various areas of SRL at its various phases. The study also attempted to identify technical features and a combination of interventions which were found to be effective for SRL support. As a result, the potential inventory of interventions was drawn up in the form of tables (see Tables 3–6).

### General Overview of the Findings of the Interventions Targeting Various Areas of Self-Regulated Learning

In the current study, the distribution of the interventions explored focusing on various SRL areas has an unbalanced nature, with metacognition regulation scaffolds being by far the most explored, whereas emotion regulation interventions are the least investigated (see Table 1 above). As for the focus on SRL phases, the overwhelming majority of the interventions target SRL areas in the performance phase, even though the planning phase is considered as the most important of the three (Greene et al., 2012; Yılmaz et al., 2017), especially for novice and less motivated learners, who need extra support, particularly at the “set up” stage. This finding is not in line with a study by Viberg et al. (2020) where the planning phase is claimed to be equally well supported. This can be explained by the fact that in this study, no differentiation is made between SRL areas and phases, and the interventions targeting preparation phase of the metacognition regulation (and which are well covered according to the present review as well), compensate for the interventions largely absent from the preparation phases of other SRL areas.

Additionally, SRL is a multi-component and complex construct; it is a cyclical process, and the activities in each phase, which are also non-linear or lacking a subsequent nature, affect one another (Zimmerman, 1990). Under supporting any given component or phase can have a disruptive effect on the whole SRL support process. Thus, “the connectedness” (Wong et al., 2019, p. 369) in the process of support among all SRL areas and phases needs to be born in mind, and the comprehensiveness of the support designs must be ensured (Greene et al., 2012). The fact that none of the support interventions explored in the current study were found to be covering all SRL areas as well as phases is in line with the above claim about its complex nature. Hence, to achieve optimal outcomes with regard to SRL support, it becomes necessary to engage in careful mixing and matching of various interventions while keeping in mind the context, the learner as well as the task characteristics at hand.

However, if a single most flexible and comprehensive intervention had to be selected, that would be prompts. As shown in the present study, prompts can come in different forms (e.g., text, pedagogical agents) and at different times (planning-, learning process- and reflection-oriented prompts). Various prompts are also presented in combination with other interventions (e.g., with the feedback) for an enhanced effect. They can be of varying levels of specificity (personalized vs general), and delivered at micro (task-level) as well as macro level (study cycle level). Prompts also vary according to the level of individualization and flexibility (e.g., adaptive/personalized prompts), learners’ involvement (e.g., self-directed prompts) and intensity of support they provide. Prompts have been found the most useful for learners with fewer skills and competence as well as for more complex tasks (Kauffman et al., 2011). However, the studies reviewed also revealed the importance of taking into account various factors to ensure the successful application of prompts in specific contexts and with specific learner groups. For instance, whereas more detailed and more frequent prompts have been found to be efficient with more inexperienced learners, prompts that are more strategic and generic in nature have been proven to work better with more experienced learners, avoiding unnecessary overload and distraction.

To broadly summarize the findings with regard to the areas of SRL that the identified interventions cover, it can be observed that cognition regulation seems to be supported at the task level in most of the cases and at the performance stage of the learning process; the findings are in line with previous studies (Devolder et al., 2012). The interventions targeting cognition regulation largely support learners with engaging more deeply with the content through being reminded to revisit the materials, ask for further clarifications, prompting and giving the tools to summarize, highlight, take notes and thus interact with the content as much as possible. Such interactive practice is in line with the claims of the learning theorists that learners should “do something” with learning materials rather than just be exposed to them and stay in the role of the passive recipient (Jonassen et al., 1998). As for metacognition, it is the most comprehensively supported and investigated SRL area. The biggest fascination with metacognition can be explained by the fact that regulation is mostly associated with planning and actual performance-related learning strategy use. Thus, the need for metacognitive support might seem more prominent. However, the importance of supporting cognition, motivation and, especially, emotion regulation, which are more associated with internal learning processes, seems to be somewhat underestimated. In the current review, motivation and emotion regulation support have been found to be closely interconnected as well as related to other areas of SRL (cognition and metacognition). In none of the studies was the motivation or emotion component the only and explicit target of the exploration but rather investigated together with other areas of SRL. This could be explained by the fact that motivation and emotion regulation, besides external and learner-related characteristics, are also largely defined by cognition and metacognition regulation as well as influencing one another (Weiner, 1985). With regard to motivation, as part of the current review, it can be further observed that motivation is investigated not only as a dependent variable but, in a couple of cases (e.g., Bannert and Mengelkamp, 2013; Duffy and Azevedo, 2015; Ilves et al., 2018), as an independent variable having its differential effect on other SRL area outcomes, indicating an excessive interdependence of motivation regulation with various aspects of self-regulated learning and underlining the necessity to look at it in combination with other areas of SRL. For instance, when learners feel totally lost facing complex content which is beyond their “reach,” then, if they are unequipped with special metacognitive strategies that would help them navigate through the online learning experience, they might find a more feasible alternative—to avoid the failure by just giving up.

The present literature review revealed a positive association of motivation and emotion regulation with goal setting and planning conducted at the preparatory phase of the SRL cycle. This observation is supported by the Goal Orientation theory, according to which goal setting is a key motivational process (Locke and Latham, 1984). Since the set goals define the ultimate outcome that individuals are trying to achieve, they are more likely to engage in activities that are believed to lead to those goals. Goals that are specific, realistic and adapted to learners’ needs are highly motivational and translate into increased learner self-efficacy at the preparatory phase. It also has the potential to reduce learners’ anxiety levels by engaging them in setting goals that seem more realistic and feasible. Further, motivation is maintained during the performance phase by learners being more prepared and less anxious about what comes next. Thus, it is no surprise that interventions such as planning and reflection tools, assessment rubrics and planning prompts, aimed at clarifying the expectations and setting out clear paths for learners to follow, have been shown to have a positive effect on motivation and emotion regulation. Even in the face of challenging tasks, knowing what to expect and what the priorities are results in reduced stress levels and increased motivation to persist in the learning process.

Unlike motivation regulation, which has been well explored at the performance phase of SRL, emotion regulation has been vastly under investigated. Fortunately, it seems that the existing gap has been identified in other studies as well (Duffy and Azevedo, 2015; Hooshyar et al., 2020), the realization of the need to integrate affective components of SRL into instructional settings are beginning to emerge (Belland et al., 2013) and more and more calls have been made to develop “systems that care” (Du Boulay et al., 2010, p. 197).

### Effective Combination of Interventions and Their Technical Features for Self-Regulated Learning Support

#### Combination of Interventions

As shown by the findings of the current review, the most optimal and feasible way to provide comprehensive support for self-regulated learning in distance learning environments is by accurately and thoughtfully combining various interventions. Additionally, it is important that each intervention is carefully crafted, paying attention to each of its feature as well as taking into account a myriad of factors emerging from the context at hand. Otherwise, potentially very powerful tools might turn into useless or even hindering measures. For instance, directed pre-flection prompts (Lehmann et al., 2014) were found to be positively affecting novice learners’ motivation, but less efficient with more advanced and experienced learners, whereas a study by Ifenthaler (2012) proves the efficiency of generic prompts with more advanced learners more pronounced (Wong et al., 2019). In the present review, generic reflection prompts used on their own without reinforcement of their effect with feedback, and used with less experienced learners, proved to have no effect on SRL (Verpoorten et al., 2012; Bannert and Mengelkamp, 2013). According to Verpoorten et al. (2012), the prompt, a potentially powerful SRL intervention, can turn into a “featherweight technique” (p. 8), unless designed efficiently and used with the right audience. Thus, it becomes very difficult to design a one-of-a-kind intervention that can “do magi” in all these cases. The solution may be attributing the online learning process a more individualized nature (see discussion below).

As for an impactful combination of SRL interventions, it was found that reflection prompts enhance the effects of planning e-journals by encouraging further reflection on learners’ part with regard to their metacognitive strategy use. Also, a note-taking tool highly benefits from add-on monitoring prompts, and the combination results in more intensive processing and analysis of the notes taken. Generic prompts benefit from being reinforced by monitoring feedback for significant effects, whereas the feedback itself has a stronger effect if delivered in the visual form. In the case of SRL, multidimensional visualizations, such as radar graphs, are of most use, and a further combination of the visual feedback and the comparison feature (see detailed discussion in the paragraph below) makes the interpretation of the results easier and more productive.

Another efficient combination of interventions identified is supplementing system delivered SRL support with tutor involvement in the support process, a practice that proves to be useful in the case of complex SRL interventions and, again, particularly with learners lacking experience in operating efficiently in online learning environments independently. Peer-to peer interaction and the provision of feedback, which is a useful SRL practice, can also be further supported by employing well-defined assessment rubrics, which are expected to secure the needed quality of the feedback given and alignment of the feedback with the learning outcomes. This is an especially useful practice in the absence of intensive teacher presence.

#### Effective Technical Features

The “empowering features,” which were found to be contributing to boosting the impact of the SRL support and, in some cases, being a critical determining factor of success, are summarized below.

##### A. Personalization, Adaptability and Learner-Directed Nature of the Interventions

Personalization and adaptability of the distance learning experience can be achieved with the help of designs of the tools and learning environments that involve learners themselves in elaborating support interventions for themselves. Such practice contributes to making learners more engaged and motivated in the learning process (Bannert et al., 2015; Lee et al., 2016), the finding which is also in line with the previous studies in this area (Poot et al., 2017). Teacher involvement in providing help on an individual basis is another possibility of such support. However, even though the latter is an efficient and personalized way of support (Azevedo et al., 2011; Torras and Mayordomo, 2011; Gikandia and Morrowa, 2016), such approach might not always be a feasible solution in the present day of massive online learning. Luckily, these days, advanced technologies offer possibilities of mediating the given challenge. In the present study, interventions described as individualized and/or adaptive were the ones largely based on system generated learning analytics and Open Learner Model technologies. Open Learner Models have great potential to transform the nature of SRL support dramatically by making it possible for the system to analyze learner behavior through log data and, in the case of clearly defined indicators for each SRL area, provide a personalized and well-timed targeted support. In the present study, such technologies helped attribute the prompts (Duffy and Azevedo, 2015), feedback (Lee et al., 2010; Wäschle et al., 2014; Ilves et al., 2018) as well as the study materials (Guerra et al., 2016) an individual/adaptive nature. Within the study environments, such systems enabled automatic re-designing of the learning format—the sequence of activities, the mode of delivery of learning practices and materials based on learning styles and preferences, pre-determined based on learners’ profiles (Souki et al., 2015).

Providing students with adaptive scaffolding in the OLM environment then also means measuring learners’ levels of SRL and providing personalized support. Accordingly, clearly defining the indicators related to concrete SRL strategies becomes necessary for the system to be able to accurately deliver targeted support to the learner. The trend of combining the measurement and support of SRL is emerging in the field of self-regulated learning, and is referred to as “the third wave” (and the most efficient) of SRL support, “when measurement and intervention come hand in hand” (Panadero et al., 2016, p.1), and help provide just the right level of support. SRL is about finding “the right balance between freedom and guidance during the learning process” (Nussbaumer et al., 2014, p. 17) after all.

##### B. Social Comparison

The social comparison feature has been explored by a number of studies as a useful feature to have in distance learning environments. The motivational and time management related benefits of social comparison were identified in the studies reviewed (Guerra et al., 2016; Jivet, 2016; Ilves et al., 2018) and this finding is also in line with the previous research (Papanikolaou, 2015). Papanikolaou observed that comparing one’s behavior to a target performance largely determines how learners react to their success or failure, and helps to identify differences in their learning process. In case of success, the comparison helps learners recognize the learning strategies they adopted and optimize their strategies. On the other hand, in case of failure, the desired state motivates learners to re-evaluate and change their strategies. However, the “dangers” of using the social comparison feature have also been emphasized. Namely, while proven to make learners more engaged in the study process, such comparison, if done on a peer-to-peer basis, might be conducive to the development of a more competitive spirit among students, which might be more acceptable in some cultural contexts than in others. As for comparisons based on other students’ navigation patterns, such practice might have a unifying effect, and prevent learners from adopting new and creative ways on the way to achieving their goal (Guerra et al., 2016). Moreover, certain forms of social comparison could put pressure on learners who are lagging behind and contribute to them giving up the course instead of encouraging them to pursue their goals.

Thus, one potential way to go about the comparison issue and to encourage mastery rather than performance orientation among learners is to focus on making the comparison more general in nature. Comparison can be made of a students’ outcomes against a standard expectation level, a ‘neutral average’ rather comparing students’ success to one another, which can have a detrimental effect on low-achieving learners, and result in increased anxiety levels. Another potential approach to help learners stay focused on their own progress rather than worry about being ‘behind’ or being demotivated by progressing too ‘far ahead’ of the others is to follow up the comparative data with the textual feedback focusing on the learner’s own improvement and individual effort as much as possible. Further, to attribute this feature an adaptable nature, it can be offered to learners on an on-demand basis, by making the function optional.

## Further Research and Limitations

The gap observed in the research of the SRL support was with regard to emotion regulation in online learning environments. It can be speculated that the reason for the under-investigation of this area might be due to its highly ‘hidden’ nature. For this reason, the ability to accurately detect the need for emotion regulation becomes important (Viberg et al., 2020). For the purpose of accurate measurement of emotion regulation a multidimensional approach has been suggested (Panadero et al., 2016), such as self-assessment, or naturalistic approaches, such as observation (if feasible), as well as facial expression analysis (Järvenoja et al., 2017), applied alongside with exploiting the potential of OLM. As for the potential of OLM, the need to define accurate indicators becomes crucial, which requires further research and evidence base.

The fact that the study is limited to looking at SRL in higher education level and distant learning context, implies that the findings of the given study cannot be automatically applied in other settings (e.g., young learners and f2f formats) and these areas require further investigation. Similarly, learner-related variables might have a differentiating effect on the efficiency of certain SRL interventions. The current review revealed that the studies investigating this aspect in a systematic and thorough manner with regard to concrete interventions are scarce and need more attention.

Additionally, the present study does not compare or explore the differentiated effects of the identified interventions in the various identified learning environments. For instance, platforms that are open and non-linear in nature and allow interaction and communication and self-directed choice of materials for learning purposes would benefit more from technology provided step-by-step scaffolding, OLM technology adoption and individualization of the learning process. In contrast, distance learning environments which serve more as repositories of knowledge and leave little space for creativity or autonomy due to their passive and straightforward nature are likely to benefit from different types of support, e.g., feedback on their level of engagement with the course material and learning performance (Jivet, 2016). Hence, further exploration with regard to how concrete interventions function in different distance learning environments would provide deeper insight and facilitate the choice of optimal interventions for concrete digital learning media.

Also, since the wider context (countries where the studies included in the current literature review were conducted) is dominated by highly developed countries (see Supplementary Appendix B), it can be assumed that we are looking at places with a high level of technological development and learners with a higher level of digital competences, and thus, the effects of the interventions explored may be of a different nature in dramatically different circumstances.

## Conclusion

**FIGURE 3:**
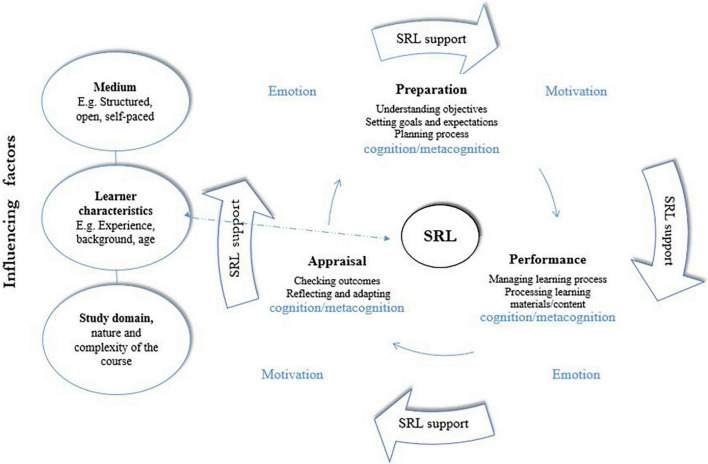
Self-regulated learning process: SRL stages and areas, strategies used, the dynamics of support provision and external factors involved.

The central aim of the present study was to investigate what interventions have been studied as part of the recent research conducted in the area of SRL, with a particular focus on distance learning and higher education level. The cyclical nature of SRL makes the comprehensive, and continuing support necessary for ultimate success. Additionally, since the SRL processes are largely determined by a number of more global, objective factors as well as learner-related characteristics, a careful account of all of these variables need to be taken into account while designing SRL support systems in online learning environments. In this direction, further, more consistent and focused research is needed for more concrete assumptions (for this reason, the relationship between these factors and SRL is presented with a dashed arrow in Figure 3). In the meantime, for the optimal and targeted learning support, the interventions that integrate personalized and adaptive features should be considered, as they were found to have the best potential to flexibly serve multiple purposes in various contexts. Customized support becomes possible with the systems that help track learner performance comprehensively, and allow adaptation of the learning process as well as more active involvement of learners themselves by giving them the access to their learning data, and allowing self-assessment and reflection. The potential of Open Learner Model systems in this direction cannot be underestimated (Hooshyar et al., 2020). Another thing to be pointed out is somewhat different nature of affective aspects (motivation and emotion) of SRL, which seem to be closely interconnected, on the one hand, as well as largely, and on an ongoing basis, affected by the factors related to the success/failure related to the cognitive and metacognitive regulation. Thus, the need for careful measurement and support of the motivation and emotion aspects of distance learning is rather pronounced. The figure below captures the above discussed points in a form of a concluding framework, which is now an expanded version of the one (see Figure 1 above) that has been used as the theoretical basis for the current study.

On a final note, amid the abundance of the SRL supportive interventions, and facing the temptation of adopting multiple technologies while trying to make the online learning environments highly supportive, it has to be born in mind that the systems and designs should stay simple, whereas the learner, their needs and the process of learning always needs to occupy the central part.

## Data Availability Statement

The original contributions presented in the study are included in the article/Supplementary Material, further inquiries can be directed to the corresponding author/s.

## Author Contributions

NE, KS, MP, and ÄL all contributed to conception and design of the study. KS took an active part in data validation, and together with MP and ÄL, to the development of the framework of analysis. NE wrote the first draft in close collaboration with other authors. KS, MP, and ÄL provided detailed feedback and contributed to the revision process of the manuscript. All authors approved the final version of the manuscript to be published.

## Conflict of Interest

The authors declare that the research was conducted in the absence of any commercial or financial relationships that could be construed as a potential conflict of interest.

## Publisher’s Note

All claims expressed in this article are solely those of the authors and do not necessarily represent those of their affiliated organizations, or those of the publisher, the editors and the reviewers. Any product that may be evaluated in this article, or claim that may be made by its manufacturer, is not guaranteed or endorsed by the publisher.

## Footnotes

1 However, these results should be taken with caution because, due to lack of relevant data, it was not possible to calculate the comparable effect size in the mentioned study.
