# Supplementary material for: Supporting Self-Regulated Learning in Distance Learning Contexts at Higher Education Level: Systematic Literature Review
Source: Front Psychol. 2022 Jan 18;12:792422. doi: 10.3389/fpsyg.2021.792422 (PMC8805682; doi:10.3389/fpsyg.2021.792422)
Supplement: Supplementary file 1 [file Table_1.pdf]

Appendix A: Intervention related terminology explained

Planning and reflection protocol (1)	A tool that allows listing learning goals before the session, at the end, reflecting on their completion, with the focus on time management/procrastination. Includes self-generated line chart visualizing learners' self-reported results.
(Directed) pre-flection prompts (2a) (2b)	Pre-flection prompts focus on planning of the learning process. 'Directed' ones are more focused and specific (e.g. to-complete-sentences with regard to the session goals).
Reflective Prompts (3a)	RPs ask learners to explain out loud the reason for the choice of the specific information node(s) by completing prompted statements (e.g. "I am choosing this page because …")
Metacognitive prompts (3b)(3c)	Pop up metacognitive prompts reminding learners of initial planning, monitoring, and evaluating their learning process.
Fading/adaptive prompts (4)	More prompts initially, and the probability of prompts being triggered decreases/increases depending on:(1) the learner compliance with the prompt, or (2) learner's metacognitive judgment inaccuracy.
Tutor provided adaptive content and process scaffolds (5)	Adaptive (1) *content scaffolding* is aimed at helping learners with checking their content understanding, (2) *process scaffolding* is aimed at helping with planning, monitoring their understanding, using different learning strategies.
Automated adaptive time management enabling system (6)	The system assists learners with their time management through adaptive release of reminders, learning monitors and learning motivators, with visual reinforcement.
Time logging tool (7)	Time tracking tool, with a possibility of visualizing the results, allowing comparison and data analysis; also, scheduling of customized reminder messages for better time management.
Mastery grids *with Social comparison feature* (8)	Intelligent interface for online learning of different types of content, which is adapted according to learners changing knowledge (through OLM), social comparison feature (peer and class progress) is included.
Pedagogical agent provided instructional prompts and feedback (9)	PA produced instruction to write a summary, assess the relevance of the content, take notes, assess understanding, re-read sections of the text; feedback provided on accuracy based on time spent viewing a particular page, and on a sub-goal(s), page relevance, number of pages visited.
Radar visualization (10)	The specific form of visualization, which allows multidimensional and multilayered presentation of information, at the same time allowing texts viewing through hovering over the values in the graph.
Matrix and outline note-taking tools (11a)	Outline and Matrix notetaking formats embedded in an online environment.
Notetaking+ *self-monitoring prompts* (11b)	SMP: brief statements inserted just prior to the sample test question that encourages learners to review their notes before moving on to the next activity.
Peer-peer formative feedback in asynchronous fora, stimulated and monitored by tutor (12)	Formative feedback provided by students to one another on the artefacts of learning they shared in a threaded asynchronous online discussion forum. Monitored and stimulated by the teacher.
Visualized feedback with social comparison (13)	Radar visualization (see above) of the aggregated information of learner past activities (collected via log traces), also allowing comparison with the peer as well as standard average.
Online platform with learner-style oriented instructional design (14)	Four learning modes corresponding to Kolb's theory: watching, discussing, conceptualizing, and trying out. This interface design gives the learner the freedom to choose the right mode(s) of learning for themselves.

Generative learning strategy prompts and metacognitive feedback (MF) (15)	Content embedded prompts ask learners to highlight important sentences in the instructional script, then summarize or organize their understanding. A pop up text in case of an incorrect answer, asking to revise.
E-portfolio based on SRL framework (16)	Open-source social networking based e-portfolio, which allows learner to engage in structure SRL phases—planning, monitoring and reflecting.
Pre-planning prompts (17)	Pop up texts asking learners to make specific plans to help engage in the course content and complete assignments on time.
Personalized e-journal + self-reflection prompts (18)	Weekly e-learning journal which includes prompts about curriculum activities, assessment tasks or learning processes in a form of questions requiring learner to react to and complete.
System, information, service quality (19)	*System quality:* usability, availability, reliability, flexibility, and adaptability<br>*Information quality*: personalized, relevant, sufficient, easy to understand, up-to-date; *Service quality*: support given by instructors or tutors.
Enhanced video tool (20)	Video learning environment, with multiple SRL support functionalities (interactivity, note-taking, highlighting/summarizing, customized supplemental resources, control buttons).
The negative impact of media diversity (21)	Online environment with diverse forms of media, which are often overlapping (e.g. videos, text, audio).
Reflection triggers (22)	3 different techniques to enact reflection: type (1)receiving information about /comparing oneself with others (2)giving information/responding about one's own learning (3)verbalizing information – by writing an annotation about their learning on each page.
Pedagogical agent-supported monitoring/reflection prompts (23)	Various questions asked by the PA at the initial stage of learning about prior knowledge, interest in the topic, plans. Weekly content unlocked, upon completion.
Group awareness tool in collaborative environment (24)	GA tool tracks information about four items: the number of personal contributions, appraisal, reply, and giving a "like"; visualizes the results of activities/interactions for learners' and instructors' analysis.
Learning Framework with adaptive presentation support technique (25)	Learners are supported to engage in setting meaningful goals, adopting appropriate strategies for managing the goals, monitoring the learning process, managing time, self-evaluating their learning paths and goals, and self-reflecting on learning outcomes.
Group awareness[1] tool (with visualized feedback) (26)	Platform tracks learners' online group activities/interactions through the GA tool, and presents cognitive, behavioral and social awareness information in real time for learner/instructor in different visual formats (pie chart, tag cloud, diagram) for observation/analysis.
System, information, service quality (27)	*System quality:* usability, availability, reliability, flexibility, and adaptability<br>*Information quality*: personalized, relevant, sufficient, easy to understand, up-to-date; *Service quality*: support given by instructors or tutors.
Help seeking prompts (28)	Written prompts indicating the importance of effective help-seeking and encouraging learners to do so, placed on the virtual workspace next to the individual tasks.
E-portfolio with techno-pedagogic design (29)	The portfolio asks of learners to engage in all 3-phases of SRL and complete relevant activities (i.e. planning, monitoring, reflection).

[1] Group awareness (GA) refers to being informed about members' learning interaction or the knowledge context (Bodemer & Dehler, 2011).

Self-assessment scripts and Assessment rubrics (30)	*Rubrics*—self-assessment tools with three components: criteria for assessing the outcome of the task, a scale for grading the different levels of achievement and a description for each qualitative level; *Scripts*—phases listed accordingly to the expert model of performing a task from beginning to end.
Self-directed metacognitive prompts (31)	Learner pre-select prompts from the list of metacognitive prompts, and configure the time stamps for their enactment in the learning process.
Instructional design workflow – PBL and SRL combined (32)	Online Instructional design based on the combination of Problem-Based Learning (PBL) and Self-Regulated Learning (SRL) principles.
