# Supplementary material for: Supporting Self-Regulated Learning in Distance Learning Contexts at Higher Education Level: Systematic Literature Review
Source: Front Psychol. 2022 Jan 18;12:792422. doi: 10.3389/fpsyg.2021.792422 (PMC8805682; doi:10.3389/fpsyg.2021.792422)
Supplement: Supplementary file 2 [file Table_2.pdf]

Appendix B: Descriptive Information about the Studies Reviewed

N	Author(s) & Year	Ctry	Learning Medium	Domain	Intervention	Design & Length	Instrument/ Source	Participants	Methods
**1a**	Wäschle et al. (2014)	DE	LMS	MED	Planning and reflection protocol	Expt. Lngt.	LG-data PR-Quest	N=18 Age: (M=24)	RM-ANOVA MANOVA
**1b**	Wäschle et al. (2014)	DE	LMS	MED	As above + *Signaling, informational, additive effects*	Expt. Lngt.	PSQ PR-Q	N=49 Age: (M=20)	HLM
**2a**	Lehmann et al. (2014)	DE	OLE	EDU	Pre-flection prompts	Expt. Cross-Sect	IMI α=0.77-0.92	N=64 Age: (M=20)	AKOVIA, ANOVA, MANOVA
**2b**	Lehmann et al. (2014)	DE	OLE	EDU	Directed pre-flection prompts	Expt. Cross-Sect	PANAVA-I α = 0.78	N=67 Age: (M=20)	AKOVIA ANOVA RP-MANOVA
**3a**	Bannert and Mengelkamp (2013)	DE	HLE	EDU	Reflective prompts*	Expt. Cross-Sect	LIST-Quest	N=46 Age: (M=24)	Ind. T-tests
**3b**	Bannert and Mengelkamp (2013)	DE	HLE	EDU	Metacognitive prompts	Expt. Cross-Sect	Video-P	N=40 Age: (M=22)	Ind. T-tests
**3c**	Bannert and Mengelkamp (2013)	DE	HLE	EDU	Metacognitive prompts + training in their use	Expt. Cross-Sect	Video-P	N:40 Age: (M=23)	Ind. T-tests
**4**	Bouchet el al. (2016)	USA	HLE	SCI	Fading/adaptive prompts	Expt. Cross-Sect	LD	N=161 Age: (M=20)	OW-ANOVA
**5**	Azavedo et al. (2011)	USA	HLE	MED	Tutor provided adaptive content and process scaffolds	Expt. Cross-Sect	Think aloud-P	N=123 Age: (M=21)	Chi-Square
**6**	Khiat, H. (2019)	SG	LMS	MED	An automated adaptive time management system	Expt. Lngt.	LD Course Stat	N=60 Age: (M=36)	Mann-Whitney Kruskal-Wallis Chi-Square

**7**	Tabuenca et al. (2015)	NL	LMS	TECH	Time logging tool	Expt. Lngt.	*LD* (α =.76-.92)	N=36 Age: (M=24)	Friedman's ANOVA
**8**	Guerra et al. (2016)	USA	Mastery Grids [1]	TECH	Mastery grids	Expt. Lngt.	LD	N=89 Age: (M=89)	OW ANOVA Kruskal-Wallis
**9**	Duffy and Azevedo (2015)	USA	HLE (multi-agent)	SCI	Instructional prompts and feedback	Expt. Cross-Sect	LD	N=84 Age: (M=21)	MANCOVA
**10**	Ilves et al. (2018)	FI	Blended online textbook	TECH	Radar and textual visualization of learner log data	Expt. Lngt.	LD	N=442 Age: (M= 442)	Wilcoxon
**11a**	Kaufman et al. (2011)	USA	OLE	EDU	Matrix and outline note-taking online tools	Expt. Lngt.	Note-taking tool Task data	N=33 Age: (M=21)	Propositional analysis Kruskal-Wallis Mann Whitney
**11b**	Kaufman et al. (2011)	USA	OLE	EDU	Note-taking tools combined with self-monitoring prompts	Expt. Lngt.	Note-taking tool Task data	N=119 Age: (M=21)	ANOVA
**12**	Gikandia and Morrowa (2014)	NZ	LMS	EDU	Peer-peer formative feedback in asynchronous forum, stimulated and monitored by the teacher	Interp. Lngt.	Observation-P Arch. Online Disc. Intw.	N=17 Age: (M=35)	Coded data Triangulation
**13**	Jivet (2016)	NL	MOOCx on LMS	TECH	Feedback with visualized social comparison feature	Expt. Lngt.	LD	N=458 Age: (M=28)	TS- Mann-Whitney U tests
**14**	Lee et al. (2016)	CAN	LMS	TECH	Online learning platform with learner-directed model	Expt. Lngt.	Observation-P Arch. OnDisc. T, S, Int	N=35 Age: (M=28)	T-test

[1] An intelligent interface for online learning content that combines open learner modeling (OLM) and social comparison features.

**15**	Lee et al. (2010)	USA	LMS	SCI	Generative learning strategy prompts and metacognitive feedback	Expt. Cross-Sect	MSLQ α =.82-96	N=223 Age: (M=21)	SEM MANCOVA
**16**	Alexiou and Paraskeva (2015)	GR	MOOCx on LMS	TECH	E-portfolio based on SRL framework	QS-Expt. Lngt.	MSLQ	N=45 Age: (N/A)	PS T-test
**17**	Yeomans and Reich (2018)	USA	LMS	SCI	Pre-planning prompts	Expt. Lngt.	Course data Surv.	N=60,778 Age: (N/A) Course based	Randomized test
**18**	Abdullah and Hashim (2019)	MY	LMS	N/A	E-journals, with self-reflection prompts	QS-Expt. Lngt.	E-journal data MSLQ α = .68- .86	N=54 Age: (N/A)	PS t-test RM-ANOVA
**19**	Albelbisi and Dina (2019)	MY	MOOC	SCI	Instructor and institutional support Course quality	Empr. Lngt.	Quest. α= .70 and .91 Fact. loading 0.6.	N=622 Age: (M=28)	PLS-SEM HTMT-Anal.
**20**	Delen et al. (2014)	USA	Video Learning Envm.	SCI	Enhanced video tool	Expt. Lngt.	SRSI α = .64-.82 LD	N=80 Age: (N/A)	IS T-test Correlations test
**21**	Lange and Costley (2019)	KR	LMS	TECH	Media diversity** (Negative effect)	Empr. Lngt.	MSQL, ICL DMT α= .77-.95	N=2363 Age: (M=35)	Correlation
**22**	Verpoorten et al. (2012)	GB	LMS	TECH	Reflection triggers	Expt. Cross-Sect	Log Data	N=54 Age: (N/A)	ANOVA
**23**	Yılmaz et al. (2017)	TR	LMS (MOODLE)	TECH	Pedagogical agent supported monitoring/reflection promt.	Expt. Lngt.	SR-Assessment Scale α = .92-.98	N=102 Age: (M=20)	ANCOVA
**24**	Lin and Tsai (2016)	TW	Online collaborat. environment	TECH	Group awareness tool	Expt. Lngt.	Log data	N=84 Age: (M=20)	ANOVA

**25**	Souki et al. (2015)	GR	LMS	EDU	Learning framework *based on learning preferences*	Expt. Lngt.	MSLQ	N=45 Age: (N/A)	PS t-test Spearman's Rho Correlation
**26**	Ma et al. (2020)	CN	LMS	TECH	Group awareness tool	QS-Expt. Lngt.	LD, LASSI, GSES α = N/A	N=165 Age: (M=19)	OW-ANOVA Paired T-test KruskalWallas
**27**	Zhao (2016)	CAN	OLE	EDU	E-learning WEB 2.0 (quality factors)	Empr. Lngt.	six-factor Quest. α =.87-.89	N=250 Age: (M=30)	CFM SEM
**28**	Schworm and Gruber (2012)	DE	LSM	EDU	Prompts on help-seeking	Expt. Lngt.	LD α = .93 Slfr-Q α = 0.66	N=39 Age: (M=23)	T-test
**29**	Torras and Mayordomo (2011)	ES	LMS	EDU	Electronic portfolio <u>+ support with its use</u>	Expt. Lngt	OP and CS (α N/A) L-T interact. data	N=17 Age: (M=27)	Descriptive Stats/Comp. stats.
**30**	Panadero et al. (2013)	ES	OLE	EDU	Rubrics and self-assessment scripts	Expt. Lngt.	EMSR-Q, Self-efficacy Q, SSR-Q (α=.78-89)	Sample: 69 Age: (M=20)	RM-ANCOVAs
**31**	Bannert et al. (2015)	DE	HLE	EDU	Self-directed metacognitive prompts	Expt. Lngt.	LD	N=70 Age: (M=20)	MANOVA Ind. T-tests
**32**	Paraskeva et al. (2017)	USA	OLE	TECH	Instructional design workflow – PBL and SRL combined	QS-Expt. Lngt.	MSLQ	N=70 Age: (M=20)	One-tailed paired t-test
**33**	Green et al. (2012)	USA	HLE	SCI	Explicit verbalization of task understanding and planning*	QS-Expt. Cross-Sect	Coding Scheme for TAP verbalizations α = N/A	N=55 Age: (M=20)	Regression

Abbreviation explanations: ***<u>Domain</u>***: MED=Medicine; EDU=Education; TECH = Technology/ICT; SCI=Science; PSY=Psychology. ***<u>Medium:</u>*** Learning Environment= LM; HLE=Hypermedia Learning Environment; LMS=Learning Management System; MOOC=Massive Open Online Course; Online Learning Environment=OLE; Online Video Based Environment=OVBE; Online Collaborative Learning Environment=OCLE. ***<u>Participants:</u>*** N=sample size; M=median age. ***<u>Study design</u>***: Cross-Sect=session-based; Exprt.=experimental; Lngt.=longitudinal (course based.); QS-Exprt=quasi-experimental design; SR=self-reported; Slfr=self-reported. ***<u>Other:</u>*** Msmt=measurement. ***<u>Instruments:</u>*** Archived Online Discourse=Arch. OnDisc.; Confirmatory Factor Model=CFM; CS=Coding System; Diversity Measurement Tool=DMT; Emotion and

Motivation Self-Regulation Questionnaire=EMSR-Q; The Heterotrait-Monotrait=HTMT; General Self-Efficacy Scale=GSES; IMI=Intrinsic Motivation Inventory; I=Instruments; Instrument for Cognitive Load=ICL; Intw=interviews; LD=log data; LG=Learner Generated; Learning and Study Skills Inventory=LASSI; O=Observation; Online learning Environment=OLE; Online Self-Regulated Learning Questionnaire=OSRQ; Partial Least Squares Structural Equation Modeling=PLS-SEM; Quest.=questionnaires; Specific Self-Regulation Questionnaire=SSR; Surv.=Survey; P=Protocols; Perceived Stress Questionnaire (PSQ) (Levenstein et al., 1993); Panava-I=PANAVA Inventory; Procrastination questionnaires (Lay and Silverman, 1996)=QR-Quest; Stat=statistics; System Usability Scale=SUS; Structural Equation Modeling=SEM; Validity Reliability of Time Management Questionnaire= VRTMQ; Video-P=Video Protocols.
Note: Interventions marked with *= had been proven to have no significant effect on SRL; **=negative effect.
